# How do online users perceive health risks during public health emergencies? Empirical evidence from China

**DOI:** 10.3389/fpsyg.2023.1087229

**Published:** 2023-01-27

**Authors:** Shijing Huang, Cheng Zhou, Qinjian Yuan, Guohua Chen, Hongzhou Shen

**Affiliations:** ^1^High-Quality Development Evaluation Institute, Nanjing University of Posts and Telecommunications, Nanjing, China; ^2^School of Public Administration, Nanjing Normal University, Nanjing, China; ^3^School of Information Management, Nanjing University, Nanjing, China; ^4^School of Management and Engineering, Nanjing University, Nanjing, China; ^5^School of Management, Nanjing University of Posts and Telecommunications, Nanjing, China

**Keywords:** public health emergencies, risk perception, information ground, construal level theory, psychological distance, grounded theory, PLS-SEM

## Abstract

**Background:**

The global COVID-19 pandemic has posed a major threat to human life and health, and new media technologies have intensified the spread of risk perception.

**Purpose:**

This study aimed to explore the impact of risk information ground on online users’ perceived health risks, and further explore the mediating role of psychological distance and the moderating role of self-efficacy.

**Methods:**

A total of 25 Internet users from different provinces in China were interviewed in-depth, NVIVO.11 was used to qualitatively analyze the interview text data and construct a theoretical model. A total of 492 interviewees were recruited in order to complete a scenario questionnaire, SPSS-27 was used to perform orthogonal experiments, generate eight combinatorial scenarios, analyze demographic data, and clean and prepare data for testing hypotheses. SmartPLS 3.0 was used to test the conceptual model using the structural equation model (SEM) of the partial least squares (PLS).

**Results:**

The analysis of the SEM model shows that all planned hypotheses (Information fluency → Information diagnosability, Information extensibility → Information diagnosability, Information diagnosability → Psychological distance, Platform interactivity → Scenario embeddedness, Network connectivity → Scenario embeddedness, Scenario embeddedness → Psychological distance, Psychological distance → Risk perception, Psychological distance → Self-efficacy → Risk perception, Information fluency → Information diagnosability → Psychological distance → Risk perception, Information extensibility → Information diagnosability → Psychological distance → Risk perception, Platform interactivity → Scenario embeddedness → Psychological distance → Risk perception, Network connectivity → Scenario embeddedness → Psychological distance → Risk perception) are confirmed.

**Conclusion:**

This study found that the information ground factors significantly affect online users’ perceptions of health risks, psychological distance mediates the effect of information ground factors on risk perception, and self-efficacy negatively moderates the effect of psychological distance on risk perception.

## 1. Introduction

The outbreak of major public health emergencies (e.g., COVID-19) poses a major threat to people’s lives and health, and also provides environmental conditions for the gathering and outbreak of social risks, especially the information explosion and fermentation in cyberspace, giving people a negative psychology of panic, rumors, superstition, and mistrust, causing serious disruption to the entire social order, and exposing society to extremely high risks (Li et al., 2020). Pandemic sociology considers emerging epidemics as a source of social instability, uncertainty, and even crisis, and holds that just as the biological environment changes as a result of epidemics, so does the social layout (Dingwall et al., 2013) and the way people learn and live (Noor et al., 2022; Younas et al., 2022). The extent of the damage caused by public health emergencies depends not only on the harm caused but also on the public’s risk perception of and reaction to the event, on how the public obtains relevant risk information, and on how it perceives and interprets such information, thereby generating corresponding risk behavior. At present, online platforms are important carriers for users to obtain and disseminate risk information about public health emergencies. In the era of full-media information, the great abundance and rapid dissemination of information make users’ online risk perceptions changeable and unpredictable. Online users’ health risk perceptions of public health emergencies are an important constraint affecting government crisis management and risk communication. Therefore, understanding the formation mechanism of users’ online risk perceptions in crisis is a prerequisite for the timely identification and prevention of other secondary social risks.

## 2. Literature review

The topic of public perception of health risks during public health emergencies has attracted the attention of many researchers in recent years (Dong et al., 2022; Ert et al., 2022), especially during the COVID-19 pandemic, which has triggered much discussion and reflection among health professionals (Attema et al., 2021).

### 2.1. Negative consequences of risk perception

Relevant studies have pointed out that the majority of members of the public may change their social group structure during such emergencies (Busby et al., 2016), and a poor sense of belonging or other mental pressures increase their degree of risk perception (Arteaga and Ugarte, 2015; Diotaiuti et al., 2022). On the one hand, risk information that has been much disseminated may contain more negative factors, deepen the fear and anxiety of individuals facing risk events (Zhao et al., 2021), and increase the probability of individuals suffering from depression and other forms of mental stress (Diotaiuti et al., 2021a,b). As a general rule, the tolerance threshold of society as a whole for risk is reduced (Bodoque et al., 2016). On the other hand, fear amplifies people’s perception of risk, which urges people to adjust their behavior, thus leading to the formation and amplification of secondary risks (Abdulkareem et al., 2018). Based on this, researchers have speculated on the possible behavioral impact of public risk perception in emergency situations from different perspectives, including the impact of risk perception on protective behavior. Grothmann and Reusswig (2006) built a sociopsychological adaptation model and believed that the variable of risk perception could better explain and predict people’s pre-disaster prevention behavior than other general factors. Knocke and Kolivras (2007) studied people’s defense behavior during flood events, pointing out that the greater people’s perception of life and health risks, the greater their probability of adopting appropriate flood defense behavior (Knocke and Kolivras, 2007). Some researchers have also discussed the impact of risk perception on information behavior. These studies have all pointed out that people’s risk perception is constantly shaped and affected in their contact and communication with others in the face of crisis events (Liu et al., 2021). The stronger people’s risk perception and the greater their concern about risk, the higher their frequency of risk communication and information searching and dissemination, which may lead to online public opinion risk (Major, 1993; Neuwirth et al., 2000).

### 2.2. Factors affecting public risk perception in public health emergencies

According to ecological theory, however, individual development is the product of interaction between individuals and the environment. Individuals in the same environment develop differently due to different individual characteristics. Not all individuals receiving the same information develop the same level of risk perception (Zhao et al., 2021). Different social groups also have different risk perception mechanisms. For example, risk experts and the general public have significantly different attitudes toward risk, and government managers maintain their own unique rules with regard to risk perception (Huang et al., 2021). In general, researchers believe that, in the context of public health emergencies, the factors affecting public risk perception are multidimensional, including:

1. *Risk characteristics*. The reason for individuals’ fear of risk is due to the characteristics of the risk itself, such as the unknown, unobservable, and uncontrollable nature of risk (Covello and Merkhoher, 1993), and the strength of the relationship between individuals and public health emergencies is positively related to the degree of perceived risk and the intensity of their reactions (Gierlach et al., 2010).

2. *Subject factors*. Byamugisha et al. (2009) pointed out that demographic factors such as an individual’s gender, age, and educational attainment level, as well as their past experiences of risky events, can affect an individual’s risk perceptions. The older the person, the lower the probability of perceiving the risk of infection, but the higher the severity of the perceived risk (Rosi et al., 2021); people who know more about the causes of diseases are more worried about being infected, that is, there is a positive correlation between knowledge and risk perception (Iorfa et al., 2020). Moreover, emotion (Wang et al., 2013; De-Juan-Ripoll et al., 2021), cognitive bias (Prentice et al., 2005; Klein et al., 2010), and sense of self-efficacy (Blanton et al., 2001; Diotaiuti et al., 2021a,b) are also important predictors.

3. *Media message factors*. The characteristics of media information itself can directly affect individuals’ risk perceptions, such as the type of information (Rotter, 1980), the amount of information (Mileti and Peek, 2000), the mode of access (Liu, 2022), and the dissemination of information (Yim and Vaganov, 2003).

4. *Governmental factors*. Studies have pointed out that the level of trust in government institutions also affects the public’s perception of risk (Yokoyama and Ikkatai, 2022). Researchers also believe that social and cultural factors affect individuals’ risk perceptions. In short, individuals’ social class, educational attainment level, values, family structure, religious beliefs, and group factors can all have an impact on risk perceptions (Liu et al., 2020).

### 2.3. Comments

A review of the relevant literature reveals that, among the “hot” research topics triggered by public health emergencies, risk perception is the core intermediary variable that triggers a series of psychological and behavioral responses in people, is widely used by researchers in the construction of both theoretical and empirical models, and is the central issue to be considered in emergency risk communication and management. The variable of risk perception is often used to explain and predict the network public opinion risk and other social risks of public health emergencies, which is also the theoretical and practical basis for risk communication and emergency management. However, in the current Internet environment, the formation of public risk perception increasingly depends on online risk information. How online users perceive the health risks of public health emergencies has become an important issue. Although existing studies have discussed the impact of various factors such as the amount, type (positive or negative), form (text and pictures) of risk information on online users’ risk perception in different circumstances, the risk information factors extracted in previous studies may not necessarily match the perceptions of health risks by Chinese online users. Therefore, although the existing literature provides a considerable theoretical basis for this study, during the recent COVID-19 outbreak in China, which risk information factors affected the perception of health risks by Chinese online users to a greater extent need to be further investigated. Many studies have regarded various risk information factors as independent variables, and did not fully pay attention to the risk information ground formed by various characteristics of online risk information itself, nor have they divided risk information types, from both cognitive and perceptual perspectives, and measured the differences between different paths of influence at the same time. Moreover, most previous studies have used risk perception as an intermediary variable to explore its impact on other behaviors or reactions, while ignoring the results of studies on the generation process of risk perception itself. Risk perception is usually set to be directly affected by external environmental factors and individual characteristics among general models, that is, only the effect of the strength of various factors on risk perception is considered, while the mechanism that psychological distance adopts in the formation of various factors on risk perception is ignored.

### 2.4. Statement of the study

The purpose of this study is to explore the factors that influence online users’ perceived health risks in the public health emergency scenario. Risk information dissemination leads to public panic during major emergencies (Tsao et al., 2019). Especially in today’s Internet era, information has the characteristics of fast and wide dissemination, which will have a more obvious impact on public psychology and behavior (Rousseau et al., 2015). According to health information risk perception theory, the characteristics of information perceived by individuals directly affect health information risk perception (Zhao and Chen, 2020). High-quality information can reduce the uncertainty of individuals facing unfamiliar information, increase the perceived value of exchanged information, and thus reduce the perception of risk. This study is important because it provides risk information publishers and policymakers with a new perspective on how to properly disseminate risk information about public health emergencies taking into account the psychological distance and self-efficacy of online users.

## 3. Qualitative study

### 3.1. Data collection and analysis

To identify the factors that influence online users’ perceived health risks in the public health emergency scenario, we conducted 25 semi-structure interviews with online users from different regions of China.

#### 3.1.1. Participants

Purposive sampling can provide the richest information for research questions. According to the 47th Statistical Report on the Development of China’s Internet, by the end of June 2020, the age structure of Internet users in China was approximately 60% of those aged 20–49, and the proportion of those aged 50 and above had increased to approximately 20%; the gender ratio was approximately 51: 49 male to female. With respect to occupational structure, the netizen group had the largest number of middle school students, accounting for approximately 23.7%, followed by ordinary professionals or self-employed people. Therefore, when selecting interviewees, this study focused mainly on college students under 45 years old and ordinary office workers. Finally, a total of 25 interviewees were recruited for this study (including 20 for rooting analysis and five for a saturation test). The basic information relating to the interviewees is listed in Table 1. It can be seen that the ratio of men to women is basically balanced. The interviewees were mainly young and middle-aged people. Their occupations included: students at school, employees of state-owned enterprises and institutions, college teachers, self-employed people, etc. Their educational backgrounds were distributed mainly in junior colleges, undergraduate courses, masters and doctors degree courses. The regions where the interviewees were located include eastern, northern, central, southern China, and other areas. The interviewees had rich experience of using Internet platforms, and were more sensitive to network risk information perception.

**Table 1:** Description of interviewees.

Demographics		Percentage (%)
Gender	Male	48
	Female	52
Age	20–29	60
	30–39	28
	40–49	12
Education	Junior college or belowe	8
	Undergraduate	36
	Master	52
	Doctor	4
Occupation	Student	28
	Staff of private enterprises	48
	Staff of state-owned enterprises	24
Area of residence	South China	8
	Central China	12
	North China	8
	East China	64
	Northeast	4
	Northwest	4
	Southwest	4
Network platform for obtaining information related to public health emergencies	Microblog	68
	Tiktok	28
	WeChat	40
	Zhihu	20
	Official government website	20
	News client	28
	Other platforms	40

#### 3.1.2. Data collection process

The first stage of data collection lasted from July to November 2020, with 15 people interviewed; in the second stage, from July to August 2021, 10 people were interviewed. Because of COVID-19 restrictions, the 40-min semi-structured interviews took place by telephone or online. Before each interview, the topic and purpose of the interview were explained to the interviewees so that they could fully understand the intention behind the interview and ensure that comprehensive information was obtained and deeper content was uncovered. Before the formal interviews, pre-interviews were conducted with three interviewees who had experience of using online social networking platforms, and the interview outline was revised and improved based on the experience and feedback of these interviewees, following which, the formal interview outline was finally drawn up, which is presented in Table 2.

**Table 2:** Interview outline.

Number	Interview questions
1	What social platforms or network applications do you usually use to learn about the COVID-19?
2	What do you think is the difference between the information provided by these platforms or applications on the COVID-19? (Information features)
3	Talk about the different feelings of these platforms when you use them to view risk information about the COVID-19? (Platform features)
4	Do you think the risk information about the COVID-19 released by these platforms will affect your risk perception? Why? Please be specific (Information features)
5	Do you think different platforms will make you have different perceptions of the risk of COVID-19 epidemic? Why? Please be specific (Platform features)
6	Which platform environment (atmosphere) will make you feel that the risk of COVID-19 infection is relatively high or low? Why? Please give examples (Platform features)
7	What kind of content released by the platform makes you think the risk of COVID-19 infection is relatively high or low? Why? Please give examples (Information features)
8	How do you think your risk of COVID-19 infection is compared with other people (greater/smaller)? Why?(Individual features)
9	What do you think of the protection ability (stronger/weaker) compared with others in taking effective measures to ensure that you are not infected by viruses? Why? (Individual features)

#### 3.1.3. Data analysis process

This study aimed to analyze interview data based Grounded theory. The Grounded theory requires the researcher to start from actual observation without theoretical assumptions, conduct repeated searches for core concepts that can reflect the research phenomenon through systematically collected data, and finally realize the construction of a theoretical model through conceptual condensation and mapping of relationships. Grounded theory analysis consists of three classical stages. The first stage is the acquisition of primary data. In this stage, the research takes the form of one-on-one in-depth interviews with open-ended repeated multiple questions to obtain first-hand raw interview data. The second stage involves data coding. In this stage, the induction and abstract extraction of the raw data needs to be completed. This study adopted the procedural rooting process proposed by Strauss and Corbin (1994), and the specific implementation process included open-ended coding, spindle coding, and selective coding. In the third stage, the theoretical model was constructed based on the coded data. Three researchers executed the coding of the data, resolving differences through continuous comparison until members reached a consensus. The data from the first 22 interviews were analyzed first, and no new categories emerged in the data from the last three interviews, indicating that theoretical saturation was reached.

### 3.2. Hypotheses and theoretical model

**Figure 1:**
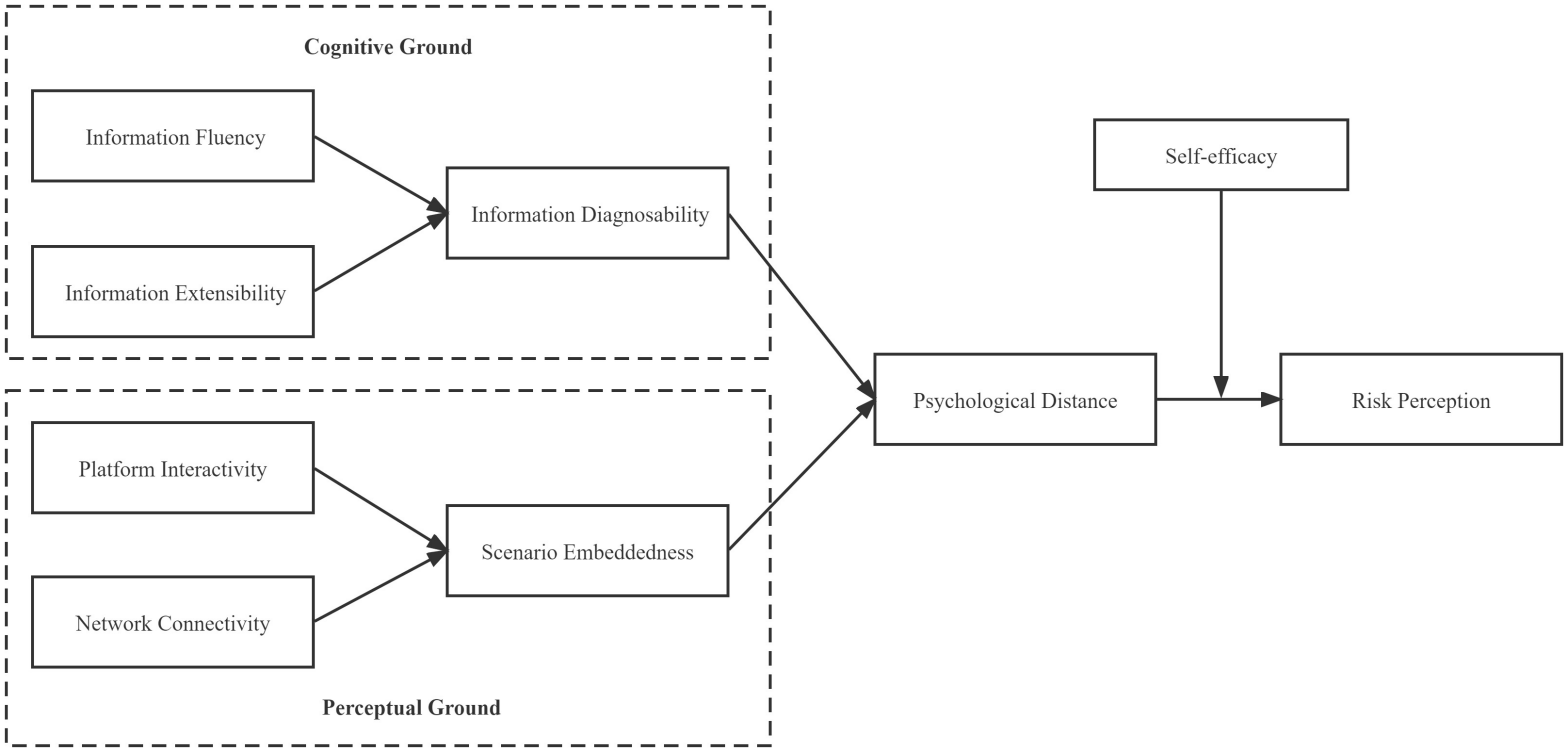
Research model.

Based on the interviews and inductive analyses, we identified eight factors that influence online users’ perception of health risks including the following: information fluency, information extensibility, platform interactivity, network connectivity, information diagnosability, scenario embeddedness, psychological distance, and self-efficacy. By analyzing and comparing the internal relationships among the categories, the main categories were refined, and the core categories were obtained based on further sorting of the main category relationships, as well as the complete “storyline” and the typical relationships between the main categories, as shown in Table 3. In this section, we develop the hypotheses and research model (Figure 1) based on our interview transcripts and previous literature.

**Table 3:** Core category and its relationship with main category.

Class	Main category	Category	Relationship
**Antecedent cause**			
Information factors	B4:Information fluency (IF)	AA10:Information acceptability	It is a component of information fluency, that is, it indicates the difficulty of individuals to obtain and process information
		AA11:Information comprehensibility	
	B5:Information extensibility (IE)	AA12:Information amount	It is an integral part of information extensibility, that is, the depth and breadth of risk information acquired by individuals
		AA13:Information comprehensiveness	
Platform factors	B7:Platform interactivity (PI)	AA15:Interaction convenience	It is a component of platform interactivity, that is, the degree of interaction convenience between individuals and platform users
		AA16:Interactive Visibility	
	B8:Network connectivity (NC)	AA17:Interaction intensity	It is a component of network connectivity, that is, the familiarity and interaction between platform users
		AA18:Interactive atmosphere	
**Mediating Effects**			
Information factors	B3: Information diagnosability (ID)	AA7:Information matching degree	It refers to the degree to which the individual perception of relevant epidemic information can meet the individual’s information needs at that time and match the information content required by the individual
		AA8:Information usefulness	It refers to whether individual perception of relevant epidemic information is helpful to individual judgment of risk de
		AA9:Information interpretation	It refers to whether the information about the epidemic scenario perceived by individuals can explain the specific problems in depth, detail and reasonably
Platform factors	B:Scenario embeddedness (SE)	AA14:Scenario embeddedness	The information atmosphere created by the platform scenes and information reporting forms enables individuals to have a sense of substitution
Individual factors	B1:Psychological distance (PD)	AA1:Probability distance	It is a component of psychological distance, that is, the distance of individual subjective perception. When people take themselves or things as reference points, they will have subjective perception of certain things at this time, here and here.
		AA2:Spatial distance	
		AA3:social distance	
		AA4:Time distance	
**Moderating Effects**			
	B2:Self-efficacy (SEF)	AA5:Epidemic prevention and response capacity	It is a component of self-efficacy, that is, the ability of people to avoid risks by relying on their own knowledge and experience
		AA6:Individual comprehensive quality	
**Results**			
Results	B9:Risk perception (RP)	AA19:Risk perception decline	It is the presentation of individual’s network perception of risk
		AA20 risk perception increase	

#### 3.2.1. Antecedent factors

Information fluency (IF) refers to how easy it is for individuals to perceive or process information. Participants in the qualitative study reported Risk information that is easy to understand enables them to identify health risks more quickly. For example, an interviewee stated: *“… it is probably the cold confirmatory number, which most intuitively gives a sense of the risk of contracting a COVID … Some animations in a more appealing form explain the transmission mechanism of this virus ….”*

Therefore, we hypothesize that:

*H1*: Information fluency has a positive impact on users’ perceived information diagnosability.

Information extensibility (IE) refers to whether the content of information is sufficiently specific, the vividness of the image, the presentation process of the details, and the quantity and richness of the information. In a time-constrained environment, individuals do not allocate more time to continue deep information processing, are more likely to accept and identify with the information they see, form risk diagnoses more quickly, and are more likely to resonate and reinforce cognitively and emotionally, leading to increased individual self-perception of risk. In the original data from the interviews, an interviewee clearly stated: *“It is better to have more information. I need to synthesize a lot of information from different aspects to judge, but the more information I read, especially in some in-depth reports, involving complex scientific knowledge, the easier it is to understand the information.”*

Therefore, we hypothesize that:

*H2*: Information extensibility has a positive impact on users’ perceived information diagnosability.

Platform interactivity (PI) refers to the immediacy and convenience of interactive communication between users and platform participants, access to information, and access to feedback. A more interactive platform makes it easier for online users to have a more realistic and contextual embedding of the outbreak information. For example, an interviewee said: *“The microblogging platform is more open and inclusive in discussing the epidemic, and I can often find real comments and feelings about the epidemic posted by individual users on the microblogging platform, so I feel a strong sense of immersion”.*

Therefore, we hypothesize that:

*H3*: Platform interactivity has a positive impact on users’ perceived scenario embeddedness.

Network connectivity (NC) refers to the strength of relationships between platform users, as evidenced by the degree of mutual familiarity, frequency of interaction, and interactive atmosphere between users on a given platform. A high frequency of interaction and a high degree of familiarity between platform users will bring people closer to this type of information, giving people a stronger sense of self-connection, and people are more likely to be influenced by this type of information. For example, one respondent mentioned that: *“Most of the people in the WeChat group are people who know each other and are familiar with each other, and people usually share some useful knowledge and also some gossip ….”* Therefore, we hypothesize that:

*H4*: Network connectivity has a positive impact on users’ perceived scenario embeddedness.

#### 3.2.2. Mediating factors

Information diagnosability (ID) refers to the extent to which it meets the information needs of online users, matches the content of the information needed, and can explain the relevant risk issues in a reasoned manner so as to help online users make judgments about the magnitude of the risk. Participant reports show that the higher the perceived diagnosability of risk by online users, the closer the psychological distance. For example, one respondent mentioned that: *“some information is detailed, including the person’s occupation, family situation, various whereabouts before diagnosis, how one becomes infected,* etc. *Seeing the process of these people being diagnosed can have an emotional impact.”*

Therefore, we hypothesize that:

*H5a*: Perceived information diagnosability has a negative impact on users’ psychological distance.

*H5b*: There is a mediating role of information diagnosability between information fluency and psychological distance.

*H5c*: There is a mediating role of information diagnosability between information extensibility and psychological distance.

Situational embeddedness refers to the extent to which online users perceive vivid and diverse reports related to the epidemic, allowing them to relate these situations to themselves and create a sense of immersion in the scenario. Situational embeddedness makes online users reduce the psychological distance to health risk. As one of the interviewees related: *“If the information on Weibo has pictures and videos, it looks like it is real, and many people are talking about it. I will tend to think that it is real, and I will warn my friends and family about it in time.”*

Therefore, we hypothesize that:

*H6a*: Scenario embeddedness has a negative impact on users’ psychological distance.

*H6b*: Scenario embeddedness mediates the relationship between platform interactivity and psychological distance.

*H6c*: Scenario embeddedness mediates between network connectivity and psychological distance.

Psychological distance (PD) is used to characterize the subjective experience of a particular event close to or far from the self, here and now (Trope et al., 2007). Some scholars have argued that many risks are perceived as a psychological distancing of the general public and that this psychological distance reduces people’s perception of risk (McDonald et al., 2015). For example, one respondent mentioned that:

*When faced with a serious scenario of epidemic prevention and control, I will pay attention to the development of the epidemic in my neighborhood and get information about the development of the epidemic in many ways, especially when I see that there is a confirmed case of the disease in a local or nearby place. Then, I will feel that the current scenario is not optimistic and I will be worried.*

Therefore, we hypothesize that:

*H7*: Psychological distance has a negative impact on online users’ risk perception.

Through the analysis of the materials, people in the epidemic will continue to receive detailed information from various sources, and in the case of a public health emergency, the longer the time, the more information obtained, and the greater the psychological ups and downs of people. There is no certainty about the risk of the epidemic. One interviewee said: *“It’s useless just to know the numbers. We only know where and how many people have recovered. It can be good and bad when it’s far away from us. We do not know whether it will be okay or not. We still have to get on with our lives”.*

Therefore, we hypothesize that:

*H8a*: Psychological distance mediates between perceived information diagnosability and risk perception.

*H8b*: Information diagnosability and psychological distance have a chain mediating effect in the process of information fluency influencing risk perception.

*H8c*: Information diagnosability and psychological distance have a chain mediating effect in the process of information extensibility influencing risk perception.

One respondent stated: *“I can see whether my place is at risk or not by using the epidemic map app, and I am relieved if the number is 0.”* The number is an abstract expression, and it shows that information with a high level of interpretation will distance individuals psychologically and reduce their risk perception. However, another respondent said: *“I think the platform with live or short videos can suddenly make me worried and anxious, and then I am more susceptible to influence …”* This indicates that high scenario embedding allows individuals to shorten the psychological distance by indenting social distance, making individuals associate events with their current scenario, thereby increasing their risk perception.

Therefore, we hypothesize that:

*H9a*: Psychological distance mediates the relationship between perceived scenario embeddedness and risk perception.

An interviewee noted that: *“when viewing the news of the epidemic, it is easier to see other people’s comments, so I unconsciously want to read more about other online users’ opinions.”* This shows that with more interaction on the platform, people can share their views and opinions through retweeting and commenting, thereby shortening the social distance between people. Closer social distance makes individuals more sensitive and cautious about risky events.

Therefore, we hypothesize that:

*H9b*: scenario embeddedness and psychological distance have a chain mediating effect in the process of platform interactivity affecting risk perception.

*H9c*: scenario embeddedness and psychological distance have a chain mediating effect in the process of network connectivity affecting risk perception.

#### 3.2.3. Moderating factors

Self-efficacy in this study is defined as the degree of confidence people have in their ability to take various types of measures to keep themselves safe from COVID infection while participating in social activities (Bandura, 1977). The interview reports show that even when online users are faced with the same risk information, they show higher levels of risk perception when their self-efficacy is low, while online users with higher self-efficacy have lower risk perceptions.

Therefore, we hypothesize that:

*H10*: Individual self-efficacy positively moderates the effect of psychological distance on risk perception.

#### 3.2.4. Result

Risk perception is the result of an individual’s multidimensional perception and experience of possible risks in the external environment and that this risk perception is closely related to life and health in the context of public health emergencies (Slovic, 1987; Wildavsky and Dake, 1990; Kahlor et al., 2010). In the current era of highly prevalent social media, the media is more likely to influence users’ risk perceptions by disseminating risk information, and the public relies more on media information for risk assessment and judgment. In this paper, since the issue of the influence of the information ground on online users’ risk perception is explored in the specific context of a public health emergency, the object of risk perception refers to the perception of health risks, specifically the assessment of the possibility and severity of one’s health being harmed by a specific public health emergency, and the concerns and fears of this health risk.

## 4. Quantitative study

### 4.1. Methodology

#### 4.1.1. Study locale

The present study has been conducted in Nanjing, the capital of Jiangsu Province in China. The online survey was used to gather primary data according to the research goals. The research goal was explained to all survey participants and their agreement was obtained. The researchers did a quality check while the data were being gathered. All the people who participated in the study were entirely voluntary, and they were told their information would only be used for research purposes.

#### 4.1.2. Study design

In order to avoid the influence of factors such as subjects’ memory and platform usage preferences, and to ensure the validity of the study data and the accuracy of the results, this study used a scenario questionnaire to collect research data.

According to the theoretical model, information fluency, information extensibility, platform interactivity, and network connectivity are all scenario variables, and can be manipulated. Based on construal level theory (CLT; Trope et al., 2007) and the results of our previous paper, it can be shown that these scenario variables were characterized by both high and low construal levels, but there are multiple levels of combination between different information ground elements. In other words, the information fluency of a specific information ground may show a high construal level, but its place characteristics, such as interactivity, may show a low construal level. Based on this, we took different levels of values for each explanatory variable at the construal level. The scenario grouping design was based on factorial design and orthogonal design methods. The four scenario variables involved in the experiment were combined according to different levels. In line with the full factor experimental design, all combinations of the factors at different levels were obtained, with a total of 2^4^ = 16 scenarios. In order to improve the efficiency of scenario design, this study used the orthogonal experimental design function of SPSS-27 software^1^ to finally generate eight combined scenarios. In the actual questionnaire distribution, scenarios were randomly assigned to the subjects. The specific scenario grouping is listed in Table 4.

**Table 4:** Design of scenario grouping.

Scenario	IF	IE	PI	NC
1	High	Low	High	Low
2	Low	Low	High	High
3	High	Low	Low	High
4	Low	High	Low	High
5	Low	Low	Low	Low
6	Low	High	High	Low
7	High	High	Low	Low
8	High	High	High	High

Eight sets of scenario materials were designed for the study scenarios, consisting of textual descriptions, pictures, and video links. After repeated rounds of small-scale studies, modification, and testing, the scenario materials were considered capable of enabling respondents to accurately perceive the high and low levels of different scenario variables. They were therefore used in the formal questionnaire. Specific scenario groupings and scenario descriptions are listed in Table 5.

**Table 5:** Scenario variable description.

Scenario variables	Level	Specific text description of the scenario
IF	High	You want to know about the epidemic scenario of the new type of coronary pneumonia. You have seen the following reports, including text, pictures or videos. The text part succinctly introduces the course and consequences of the event, and the writing is smooth. The number of infected people and the location of the incident and other important information are clear; Pictures and videos provide more detailed information.
	Low	You want to know about the epidemic scenario of the new type of coronary pneumonia. You can see the following report. The text organization of the report is quite lengthy, which takes a long time to read. The key figures and locations need to be carefully searched to determine. There are some professional terms that make it difficult to understand.
IE	High	You want to know more about the epidemic scenario of this new type of coronary pneumonia. The pictures or videos provide a more detailed introduction. In addition, the titles and network links of more relevant information are also provided to help you further explore.
	Low	You want to know more about the epidemic scenario of this new type of coronary pneumonia, but there is no more information to show.
PI	High	You want to check the views of other network users on the new coronary pneumonia epidemic. It is found that the platform has a user comment function. You can easily see the comments of other users and publish your own views. At the same time, you can also like or forward the information and communicate with other friends.
	Low	You want to check the views of other network users on this new type of coronary pneumonia epidemic, but you find that the platform has not opened the user comment function, and you can neither see nor comment on other users’ comments.
NC	High	You often use this platform to communicate with others, and your family and friends are using this platform.
	Low	You rarely use this platform to communicate with others, and your family and friends rarely use this platform.

#### 4.1.3. Sampling

The sample size for this research was *N* = 468, which was chosen using purposive sampling. The criteria were online users who are proficient in using Internet platforms to access health risk information about COVID-19.The information gathered during data collection was divided into categories depending on the frequency and percentages of each question in demographics, and Table 6 summarizes the findings.

**Table 6:** Sample description and statistical results.

Content	Category	Frequency	Percentage (%)
Gender	Male	231	49.36
	Female	237	50.64
Age	Under 18	5	1.07
	18–25	203	43.38
	26–30	150	32.05
	31–40	101	21.58
	41–50	7	1.5
	Over 50	2	0.43
Education level	High school and below	13	2.78
	Higher vocational colleges	42	8.97
	Undergraduate college	347	74.15
	Master or above	66	14.1
Have you personally experienced the COVID-19	Yes	44	9.4
	No	424	90.6
Platforms for understanding information related to public health emergencies	Micro-blog	306	65.38
	News client	240	51.28
	Baidu and other search platforms	54	11.54
	WeChat and other social platforms	218	46.58
	Zhihu question and answer platform	120	25.64
	Short video platforms such as Tiktok	121	25.85
	Other	10	2.14

There were 231 male and 237 female among the responders. A total of 208 respondents were under 26 years, 150 were between the ages of 26 and 30 years, 101 were between the ages of 31 and 40 years, nine respondents were over 41 years old. Thirteen respondents were high school graduates or below, 42 respondents had a college degree, 347 were from bachelor’s degree, 66 were from master’s degree or above. About 65% of respondents use micro-blog to get information about public health emergencies, more than half use news clients, and about 46% use WeChat or other social networking platforms.

The sample distribution of each scenario is presented in Table 7. Each scenario has at least 55 samples, and the sample distribution was relatively uniform. A one-way analysis of variance (ANOVA) test was performed in order to test the variability of the demographic characteristics between the different scenario groups to ensure the validity of the subsequent hypothesis testing. According to Table 8, the aspects of gender, age, educational attainment level, and whether the subject or people around them were infected with COVID-19 were not significantly different at the 0.01 level, which means that there were no significant differences between the sample demographic characteristics of each scenario grouping. Next, a one-way ANOVA analysis of each variable was performed with the aim of testing whether the scenario subgroups had a significant effect on the study variables. The test results showed that all study variables in each subgroup were significantly different at the 0.01 level, indicating that the scenario grouping was valid.

**Table 7:** Sample distribution.

Scene	Number of samples	Proportion of samples	Scene	Number of samples	Proportion of samples
1	55	11.75%	5	58	12.39%
2	59	12.61%	6	60	12.82%
3	57	12.18%	7	57	12.18%
4	63	13.46%	8	59	12.61%

**Table 8:** One-way ANOVA results of demographic characteristics of each scenario group.

Demographic characteristics		Gender	Age	Education level	Experience or not	Significance
Grouping (mean ± standard deviation)	1.0 (*n* = 55)	1.45 ± 0.50	2.74 ± 0.80	3.02 ± 0.47	1.98 ± 0.15	*p < 0.05 **p < 0.01
	2.0 (*n* = 59)	1.49 ± 0.51	2.82 ± 0.85	3.00 ± 0.56	1.90 ± 0.31	
	3.0 (*n* = 57)	1.46 ± 0.51	2.95 ± 0.97	2.95 ± 0.76	1.85 ± 0.37	
	4.0 (*n* = 63)	1.47 ± 0.51	2.79 ± 0.91	2.82 ± 0.69	1.92 ± 0.27	
	5.0 (*n* = 58)	1.47 ± 0.51	2.89 ± 0.95	2.84 ± 0.68	1.97 ± 0.16	
	6.0 (*n* = 60)	1.50 ± 0.51	2.88 ± 0.85	3.02 ± 0.58	1.98 ± 0.16	
	7.0 (*n* = 57)	1.54 ± 0.51	2.90 ± 0.99	3.00 ± 0.61	1.82 ± 0.39	
	8.0 (*n* = 59)	1.49 ± 0.51	2.76 ± 0.80	2.97 ± 0.60	1.89 ± 0.31	
*F*		0.254	0.395	0.921	1.563	
*p*		0.993	0.958	0.519	0.107	

#### 4.1.4. Data collection

The measurement items of the scale were based on the scale information used in the existing domestic and international literature, and the scale suitable for this study was designed by combining the results of open coding in the rooting analysis of this paper. A 5-level Likert scale was used to measure each latent variable, with response options of “1” (strongly disagree) to “5” (strongly agree). Demographic variables such as gender, age, education, and experience have been shown to have an effect on individual risk perception. Therefore, to control for the potential role of these variables on individual perception of risk in major public health emergencies, gender, age, education, and any experience of major public health emergencies were selected as control variables.

In this study, a pilot study was conducted before the formal distribution of the questionnaire. A total of 45 pre-survey questionnaires were collected during the pre-survey stage. Based on the results of the pre-survey, the scenario materials and individual measurement items of the questionnaire were modified and improved, the final questionnaire is shown in Supplementary Appendix 1.

The questionnaires were produced and distributed by Questionnaire Star. A total of 492 questionnaires were distributed to the target research groups through social media platforms such as WeChat, QQ, and Pinning between July 25 and August 20, 2021. Participants received a link to the website and were invited to participate freely in the study by answering the online questionnaire. Each participant was randomly assigned to one of eight situational experimental groups. Participants saw the material display matching the situation, including text descriptions and picture information. After reading these background materials, they answered the questions in the scale.

After eliminating invalid questionnaires, 468 valid forms remained, with a validity rate of 95.1%.

#### 4.1.5. Operationalization of study variables

The questionnaires included nine variables to gather data, and 41 items were included in the questionnaires. The study’s conceptual framework contained four independent variables (such as information fluency, information extensibility, platform interactivity, and network connectivity), three mediators (such as information diagnosability, scenario embeddedness, and psychological distance), one moderator (such as self-efficacy) and one dependent variable (such as risk perception).

### 4.2. Results

#### 4.2.1. Measurement model

##### 4.2.1.1. Descriptive statistics of measurement indicators

SPSS-27 was used to describe and statistically analyze the measurement items, and the normality of each indicator was tested. The specific data are listed in Table 9. It can be seen that the data distribution is not normal. Therefore, when selecting the analysis method for the data analysis stage, we considered selecting the structural equation model (SEM) of the partial least squares (PLS) method, and used SmartPLS3.0 software^2^ to test the model.

**Table 9:** Descriptive statistical results of measurement indicators.

Variables	Measurement items	Average value	Standard deviation	Skewness	Kurtosis	Kolmogorov-Smirnov test		Shapro-Wilk test	
						D	P	W	P
IF	IF1	3.207	1.395	−0.027	−1.335	0.181	0.000**	0.876	0.000**
	IF2	3.22	1.402	0.067	−1.547	0.267	0.000**	0.83	0.000**
	IF3	3.049	1.42	0.26	−1.351	0.229	0.000**	0.844	0.000**
	IF4	3.141	1.44	0.088	−1.47	0.235	0.000**	0.851	0.000**
IE	IS1	2.936	1.322	−0.021	−1.374	0.251	0.000**	0.864	0.000**
	IS2	2.947	1.328	−0.017	−1.377	0.25	0.000**	0.864	0.000**
	IS3	2.885	1.269	0.079	−1.101	0.185	0.000**	0.906	0.000**
	IS4	3.291	1.405	−0.35	−1.334	0.287	0.000**	0.841	0.000**
ID	ID1	3.13	1.377	−0.167	−1.181	0.17	0.000**	0.892	0.000**
	ID2	3.209	1.277	−0.025	−1.119	0.162	0.000**	0.899	0.000**
	ID3	3.269	1.287	−0.149	−1.197	0.2	0.000**	0.891	0.000**
PI	PI1	3.56	1.444	−0.752	−0.848	0.291	0.000**	0.805	0.000**
	PI2	3.543	1.341	−0.525	−1.05	0.253	0.000**	0.849	0.000**
	PI3	3.382	1.278	−0.349	−1.03	0.222	0.000**	0.888	0.000**
SE	PE1	3.246	1.278	−0.097	−1.122	0.165	0.000**	0.9	0.000**
	SE2	3.308	1.362	−0.115	−1.343	0.191	0.000**	0.873	0.000**
	SE3	3.355	1.375	−0.171	−1.354	0.188	0.000**	0.866	0.000**
	SE4	3.361	1.376	−0.184	−1.354	0.189	0.000**	0.866	0.000**
NC	NC1	3.041	1.196	−0.018	−1.146	0.22	0.000**	0.89	0.000**
	NC2	3.039	1.223	0.011	−1.208	0.228	0.000**	0.884	0.000**
	NC3	2.94	1.294	0.266	−1.375	0.315	0.000**	0.825	0.000**
	NC4	2.853	1.339	0.266	−1.341	0.296	0.000**	0.845	0.000**
PD	PD1	3.643	1.044	−0.424	−1.014	0.292	0.000**	0.831	0.000**
	PD2	3.878	0.955	−0.524	−0.634	0.249	0.000**	0.851	0.000**
	PD3	3.667	1.178	−0.664	−0.305	0.208	0.000**	0.87	0.000**
	PD4	3.748	1.02	−0.329	−1.01	0.219	0.000**	0.863	0.000**
	PD5	3.737	0.965	−0.272	−0.894	0.221	0.000**	0.871	0.000**
RP	RP1	2.673	1.301	0.296	−1.201	0.251	0.000**	0.872	0.000**
	RP2	2.938	1.408	0.096	−1.355	0.211	0.000**	0.88	0.000**
	RP3	2.897	1.306	0.121	−1.081	0.168	0.000**	0.905	0.000**
	RR4	2.774	1.299	0.415	−1.088	0.28	0.000**	0.864	0.000**
SEF	SE1	3.635	0.823	−0.136	−0.5	0.25	0.000**	0.867	0.000**
	SE2	3.239	0.931	0.018	−1.092	0.237	0.000**	0.853	0.000**
	SE3	3.041	1.057	−0.07	−1.215	0.258	0.000**	0.85	0.000**
	SE4	3.107	1.023	0.291	−1.041	0.219	0.000**	0.864	0.000**
	SE5	2.942	1.003	0.359	−1.25	0.292	0.000**	0.806	0.000**
	SE6	3.47	0.949	−0.14	−0.933	0.246	0.000**	0.871	0.000**
	SE7	3.254	0.924	0.112	−0.941	0.209	0.000**	0.868	0.000**
	SE8	3.105	0.981	−0.198	−0.734	0.219	0.000**	0.89	0.000**
	SE9	3.382	0.896	−0.2	−0.906	0.27	0.000**	0.852	0.000**
	SE10	2.9	1.062	0.029	−0.926	0.21	0.000**	0.896	0.000**

** *p* < 0.01.

##### 4.2.1.2. Reliability and validity test

The test of internal consistency in this study used the Cronbach coefficient in combination with validated factor analysis to assess the internal structure of the scale. As Table 10 presents, the Cronbach coefficients of the nine constructs presented in this study were all greater than 0.9, and the factor loadings of most of the measured variables were above 0.9. The factor loadings of SE1 and SE6 were 0.796 and 0.796, respectively, which is generally acceptable insofar as the factor loadings were greater than 0.7. In short, the scales in this study had good reliability.

**Table 10:** Results of reliability and validity tests.

Variables	Cronbach’s α	CR	AVE	Square root of AVE
IF	0.993	0.991	0.982	0.965
IE	0.924	0.927	0.876	0.768
ID	0.949	0.986	0.98	0.96
PI	0.978	0.957	0.939	0.881
SE	0.991	0.983	0.968	0.937
NC	0.968	0.973	0.95	0.902
PD	0.959	0.96	0.911	0.831
RP	0.963	0.964	0.933	0.87
SEF	0.966	0.968	0.868	0.754

Structural validity is generally judged by calculating the convergent validity and discriminant validity values. The CR values of all the constructs in this paper exceeded 0.9, as is clear from Table 11, and the AVE values exceeded 0.7, indicating that the convergent validity of the measures was good and could satisfy further analysis. Comparing the open-square value of AVE with its lower correlation coefficient value shows that the open-square value of AVE was always greater than the correlation coefficient value. Therefore, the discriminant validity passed the test.

**Table 11:** Results of discrimination validity test.

	IF	IE	ID	PI	SE	NC	PD	PR	SEF
IF	0.982								
IE	−0.256	0.876							
ID	0.043	0.048	0.980						
PI	−0.096	0.077	0.185	0.939					
SE	0.121	0.271	−0.502	−0.006	0.968				
NC	−0.281	0.238	−0.108	0.419	0.092	0.950			
PD	−0.053	−0.014	0.074	−0.038	0.317	0.265	0.911		
PR	0.010	−0.085	0.108	0.254	0.189	0.144	0.470	0.933	
SEF	−0.193	0.210	0.010	−0.092	−0.238	0.156	−0.236	−0.520	0.868

##### 4.2.1.3. Common method bias test

The nine extracted common factors explained 90.854% of the total variance. Moreover, nine factors had characteristic roots greater than 1, and the percentage of cumulative variance explained by the first factor was 26.124%, less than the critical value of 40%, indicating that there was no significant common method bias in the measurement and there was no serious impact on the validity of the results.

##### 4.2.1.4. Model fitting evaluation

The indicators used by SmartPLS to evaluate the model fit were *R*^2^ and *Q*^2^, with *R*^2^ measuring the extent to which the endogenous latent variables could be explained and *Q*^2^ measuring the predictive power of the model. The fitting results of the model are listed in Table 12. The *R*^2^ of the endogenous latent variables presented in this model were all greater than 0.3, and the *R*^2^ of risk perception reached 0.449, indicating that the model had good explanatory strength. The *Q*^2^ of each endogenous latent variable was greater than 0, indicating that the research model used in this study had some predictive validity.

**Table 12:** Model fit evaluation.

Variable	*R* ^2^	Adjusted-*R*^2^	RMSE	MAE	*Q* ^2^
ID	0.314	0.310	0.953	0.778	0.19
PD	0.431	0.428	0.820	0.682	0.333
SE	0.323	0.320	0.830	0.678	0.316
RP	0.525	0.518	0.865	0.756	0.257

#### 4.2.2. Structural equation model

The significance of the path coefficients was calculated using bootstrapping.

The standard beta was utilized to determine the significance of the hypotheses, and the beta value indicates how distinct variables may differ. The hypothesized research model was used to obtain the standardized beta (*β*) value for each connection. The importance of endogenous latent variables will be judged crucial if beta (*β*) values are large and significant. The importance of each path’s beta value was determined using T-statistics and *p*-value.

##### 4.2.2.1. Direct effects

The direct effect relationships between the variables in the model were tested. Overall, as Table 13 shows, 6 of our hypotheses are supported and significant at the *p* < 0.01 level, one hypotheses is supported and significant at the *p* < 0.05 level. Our results highlight information fluency and information extensibility may enhance information diagnosability (H1, *β* = 0.136, *t* = 2.271, *p* < 0.05; H2, *β* = 0.267, *t* = 4.631, *p* < 0.01), platform interactivity and network connectivity may enhance scenario embeddedness (H3, *β* = 0.276, *t* = 5.527, *p* < 0.01; H4, *β* = 0.354, *t* = 6.907, *p* < 0.01), information diagnosability and scenario embeddedness have negative impact on users’psychological distance (H5a, *β* = −0.182, *t* = 6.857, *p* < 0.01; H6a, *β* = −0.635, *t* = 19.773, *p* < 0.01), and psychological distance has a negative impact on online users’ risk perception (H7, *β* = −0.408, *t* = 10.689, *p* < 0.01).

**Table 13:** Results of direct effect test.

Hypotheses	Path coefficients	Sample means	Standard deviation	T-statistics	*p***-**Value
H1	0.267	0.269	0.058	4.631	0.000**
H2	0.136	0.139	0.06	2.271	0.024*
H3	0.276	0.274	0.05	5.527	0.000**
H4	0.354	0.357	0.051	6.907	0.000**
H5a	−0.182	−0.183	0.027	6.857	0.000**
H6a	−0.635	−0.633	0.032	19.773	0.000**
H7	−0.408	−0.404	0.038	10.689	0.000**

* *p* < 0.05.

** *p* < 0.01.

##### 4.2.2.2. Mediating effects

For the test of multiple mediating effects, the bootstrapping method was used to test the total indirect and specific indirect effects of the mediating effects. The results of the test are compiled and summarized in Table 14.

**Table 14:** Results of mediating effect test.

Hypotheses	Indirect effect	T-statistics	*p***-**value	Direct effect	T-statistics	*p***-**value	Total effect	T-statistics	*p***-**value	Types
H5b	−0.025	2.120	0.034*	0.076	2.372	0.014*	0.051	1.583	0.095	Partial mediation
H5c	−0.042	2.617	0.009**	0.044	1.024	0.323	0.002	0.055	0.957	Full mediation
H6b	−0.141	5.485	0.000**	0.214	6.299	0.000**	0.073	1.623	0.097	Partial mediation
H6c	−0.175	6.205	0.000**	−0.558	13.420	0.000**	−0.733	21.821	0.000**	Partial mediation
H8a	0.138	4.507	0.000**	−0.269	6.755	0.000**	−0.131	2.317	0.029	Partial mediation
H8b	0.020	2.079	0.038*	−0.184	3.801	0.000**	−0.265	4.733	0.000**	Partial mediation
H8c	0.034	2.596	0.005**	−0.048	0.940	0.366	−0.116	1.897	0.058	Full mediation
H9a	0.407	8.798	0.000**	−0.407	6.284	0.000**	0.001	0.010	0.992	Partial mediation
H9b	0.114	4.835	0.000**	0.308	7.537	0.000**	0.135	2.984	0.004**	Partial mediation
H9c	0.142	5.820	0.000**	−0.277	5.635	0.000**	0.175	3.351	0.002**	Partial mediation

* *p* < 0.05.

** *p* < 0.01.

H5b was supported because information fluency had a significant direct effect on psychological distance (*β* = 0.076, *t* = 2.372, *p* < 0.05), the mediating effect of information diagnosability on information fluency and psychological distance was significant (*β* = −0.025, *t* = 2.120, *p* < 0.05). H5c also was supported where the mediating effect of information diagnosability on information extensibility and psychological distance was significant (*β* = −0.042, *t* = 2.617, *p* < 0.01); however, the direct effect of information extensibility on psychological distance was not significant, and it could be inferred that information diagnosability completely mediated the impact of information extensibility on psychological distance. H6b was supported because platform interactivity had a significant direct effect on psychological distance (*β* = 0.214, *t* = 6.299, *p* < 0.01), and the mediating effect of scenario embeddedness on platform interactivity and psychological distance was significant (*β* = −0.141, *t* = 5.485, *p* < 0.01). H6c was supported because network connectivity had a significant direct effect on psychological distance (*β* = −0.558, *t* = 13.420, *p* < 0.01). Moreover, scenario embeddedness mediated the relationship between network connectivity and psychological distance (*β* = −0.175, *t* = 6.205, *p* < 0.01).H8a and H9a were supported because the mediating effect of psychological distance on information diagnosability and risk perception was significant (*β* = 0.138, *t* = 4.507, *p* < 0.01); the mediating effect of psychological distance on scenario embeddedness and risk perception was significant (*β* = 0.407, *t* = 8.798, *p* < 0.01). Information diagnosability had a significant direct effect on risk perception (*β* = −0.209, *t* = 6.755, *p* < 0.01), scenario embeddedness had a significant direct effect on risk perception (*β* = −0.407, *t* = 6.284, *p* < 0.01), and thus it could be inferred that psychological distance played significant mediating roles in the multivariate relationships. Information fluency and information extensibility were chain mediated by information diagnosability and psychological distance in the process of affecting risk perception (*β* = 0.020, *t* = 2.079, *p* < 0.05; *β* = 0.034, *t* = 2.596, *p* < 0.01), and the total effect of information fluency on risk perception and information extensibility on risk perception was significant, but the direct effect of information extensibility on risk perception was not significant. It could therefore be inferred that H8b and H8c were both valid. Platform interactivity and network connectivity were chain mediated by scenario embeddedness and psychological distance in the process of affecting risk perception (*β* = 0.114, *t* = 4.835, *p* < 0.01; *β* = 0.142, *t* = 5.820, *p* < 0.01), and the total effect of platform interactivity on risk perception and network connectivity on risk perception was significant; therefore, it could be inferred that H9b and H9c were both valid.

##### 4.2.2.3. Moderating effect

The analysis of the moderating effect was first based on the analysis of the main effect, and the direction of the moderating effect could be determined only after the direction of the main effect influencing relationship was determined. The original hypothesis of this study pointed out that psychological distance negatively affected users’ risk perceptions. Having tested this hypothesis, we obtained data to support it. According to the process of moderating effect analysis, as psychological distance and risk perception are negatively related, self-efficacy and risk perception were also negatively related. Therefore, this study concluded that users’ self-efficacy could play a positive moderating role in the relationship between psychological distance and risk perception. We proposed hypothesis H10 to investigate the role of different levels of self-efficacy on the degree to which psychological distance affected risk perception. We tested this hypothesis, and the results showed that, after adding the moderating effect, the path test was passed (*β* = −0.209, *t* = 5.737, *p* < 0.01), indicating that the moderating effect was significant and, simultaneously, the main effect of psychological distance on risk perception was significant; the risk perception’s *R*^2^ rose to 0.449 compared with when not including moderating variables, indicating that the overall explanatory power of the model was higher with inclusion of the moderating effect of self-efficacy.

## 5. Discussion and implications

### 5.1. Discussion

COVID-19 threatens people’s physical and psychological health (Peng et al., 2022). This study investigates the factors affecting online users’ perceived health risks in China during COVID-19, and all hypotheses were proven valid.

Cognitive processing theory states that different cognitive activities imply different levels of difficulty for individuals (Jacoby et al., 1989), lower levels of fluency trigger individuals’ fine-tuned processing of stimuli and require more cognitive resources from the cognizer, whereas higher levels of fluency trigger individuals’ heuristic processing, which does not require individuals to consume excessive cognitive resources and allow for easier processing of information in a short period time (Kruger and Evans, 2004). Information with higher fluency will reduce individual cognitive load, improve individual information processing efficiency, help individuals form risk perceptions faster, reduce the psychological distance (McDonald et al., 2015) and enhance their perceptions of the diagnosability of information (Korfiatis et al., 2012; Karimi and Wang, 2016). According to the result of the SEM model, these hypotheses (H1: Information fluency → Information diagnosability, *β* = 0.136, *t* = 2.271, *p* < 0.05; H5b: Information fluency → Information diagnosability → Psychological distance, *β* = −0.025, *t* = 2.120, *p* < 0.05; H7: Psychological distance → Risk perception, *β* = −0.408, *t* = 10.689, *p* < 0.01; H8b: Information fluency → Information diagnosability → Psychological distance → Risk perception, *β* = 0.020, *t* = 2.079, *p* < 0.05) are confirmed.

The demand for information is a reflection of the online users’ demand for complete openness and transparency of information at the beginning of the epidemic. The individual believes that more information can effectively help them make judgments, and at the same time, the individual clearly recognizes that the perception and judgment of risks in the process of information processing requires individuals to improve their discernment and comprehensive understanding of the vast amount of information. The richer the information, the more conducive it is to the perception and memory of the information receiver, which affects an individual’s risk perception (Byron et al., 2018). Our study confirms these points (H2: Information extensibility → Information diagnosability, *β* = 0.267, *t* = 4.631, *p* < 0.01; H5c: Information extensibility → Information diagnosability → Psychological distance, *β* = −0.042, *t* = 2.617, *p* < 0.01; H8c: Information extensibility→ Information diagnosability → Psychological distance→ Risk perception, *β* = 0.034, *t* = 2.596, *p* < 0.01).

Studies have shown that the presence of interactive features on platforms can effectively increase the sense of user presence on commercial websites (Vendemia, 2017), and the reviewability and permanence of content allow users to repeatedly view and communicate across time and space (Bucy, 2004). Therefore, good interactivity enhances communication between users and information publishers as well as other users, and will quickly increase the psychological distance between users and generate empathy. Our findings suggest that platform interactivity can influence users’ perceptions of health risks (H3: Platform interactivity → Scenario embeddedness, *β* = 0.276, *t* = 5.527, *p* < 0.01; H6b: Platform interactivity → Scenario embeddedness → Psychological distance, *β* = −0.141, *t* = 5.485, *p* < 0.01; H9b: Platform interactivity → Scenario embeddedness → Psychological distance → Risk perception, *β* = 0.114, *t* = 4.835, *p* < 0.01).

Network connectivity refers to the frequency of user interactions in a platform and is mainly characterized by a higher frequency of interactions, emotional intimacy, and reciprocity (Sparrow et al., 2001). The study shows that if the internal network connectivity among members is stronger, it is more likely to generate stable emotional connections and higher internal trust. In addition, based on higher quality internal relationships, information flow between members is more frequent, and it is easier to pass on hidden personal information (Kanter, 2009). The more frequent the interaction between users on the platform, the more familiar the users are with each other, then the users will have a stronger sense of connection with each other and will be more susceptible to the risk information on the platform, as the results of our empirical study demonstrate (H4: Network connectivity → Scenario embeddedness, *β* = 0.354, *t* = 6.907, *p* < 0.01; H6c: Network connectivity → Scenario embeddedness→ Psychological distance, *β* = −0.175, *t* = 6.205, *p* < 0.01; H9c: Network connectivity → Scenario embeddedness → Psychological distance → Risk perception, *β* = 0.142, *t* = 5.820, *p* < 0.01).

The result of H5a (Information diagnosability → Psychological distance, *β* = −0.182, *t* = 6.857, *p* < 0.01) indicates information diagnosability may negatively affect a user’s psychological distance to perceiving health risk. This means that when online users are dealing with risk information related to public health emergencies, the more the risk information they perceive matches their needs and helps them identify and judge risks, the more likely individuals are to have a closer psychological distance to physical risks; in contrast, low diagnostic risk information makes users more likely to have a greater psychological distance. The result of H6a (Scenario embeddedness → Psychological distance, *β* = −0.635, *t* = 19.773, *p* < 0.01) reveals scenario embeddedness has a significant negative impact on psychological distance, which is consistent with the findings of previous studies (Lerche et al., 2018). High interactivity can enhance communication between users and information publishers, as well as other users. However, different from the findings of previous studies, we found that platform interactivity and network connectivity have a different impact on the psychological distance; compared with platform interactivity, network connectivity had a greater impact, which indicated that the interaction intensity and atmosphere between platform users could make it easier for users to embed scenarios, thus narrowing the psychological distance.

Through SEM examination of the overall model, we found that, in the context of public health emergencies in China, various elements of the risk information ground indirectly affect the perception of health risks through individual psychological distance. By comparison, under the condition of controlling the diagnostic ability and psychological distance of information, information extensibility has a greater impact on individual risk perception (H8a: Information diagnosability → Psychological distance → Risk perception, *β* = 0.407, *t* = 8.798, *p* < 0.01; H9a: Scenario embeddedness → Psychological distance → Risk perception, *β* = −0.407, *t* = 6.284, *p* < 0.01). This also shows that, compared with the comprehensibility of information, a large amount of rich information about public health emergencies can cause online users to have a higher risk perception, platform interaction, and network connectivity to have a higher impact on individual risk perception than information fluency and information extensibility.

Self-efficacy strengthens the negative impact of psychological distance on risk perception. Previous studies have confirmed that self-efficacy affects users’ processing of risk information, thus negatively affecting individuals’ risk perception (Zhang et al., 1999). Some researchers have used self-efficacy as an intermediary factor to predict the role of media use on risk perception (Engelman et al., 2017; Gao, 2021). Different from these studies, this study regards self-efficacy as a moderating factor due to the fact that in the pre-interview we found different individuals showed significant differences in risk perception against the same or similar risk information background, and the significant differences in their reported self-efficacy attracted our attention. The result of H10 reveals the moderating effect of self-efficacy. The psychological distance of individuals with low self-efficacy had a greater negative impact on risk perception than that of individuals with high self-efficacy. With increasing psychological distance, the risk perception of individuals with high self-efficacy reduces more quickly than that of individuals with low self-efficacy. For users with the latter, psychological distance has less impact on their perceived risk. At the same level of psychological distance, the risk perception level of individuals with low self-efficacy is significantly higher than that of individuals with high self-efficacy.

### 5.2. Implications

In order to guide online users to form rational risk perceptions in public health emergencies, the psychological distance of individuals from risk can be influenced by rationally publishing online risk information and managing the features of online platforms to adjust their perceptions of risk. This psychological distance can originate from two directions: information features and place features. From the perspective of information features, we should first pay attention to the role of information diagnosability. We have noticed that, for online users in specific situations, individuals pay more attention to the quality of information than to its quantity. Whether the information about risks is clearly defined, has sufficient depth of interpretation of risks, or is useful for users to judge risks, affects the psychological distance of individuals to those risks. On this premise, information providers should give priority to ensuring the quantity and richness of information in order to meet the information needs of online users, followed by the readability and understandability of that information, that is, the smoothness of information. From the perspective of site characteristics, we have noticed that, as an edge path, information platform features play a greater role in individual risk perception, and are the key source of individual “magnifying” or “narrowing” risk perception. Therefore, different types of risk information need to be matched with appropriate release platforms. In addition, the moderating role of self-efficacy needs to be emphasized, especially in the early stages of public health emergencies. By providing rich and effective self-help health information to help users build confidence in the face of risks, on the one hand this can reduce the risk amplification effect of users being flooded with a large quantity of risk information, and on the other hand can also help users establish a rational attitude toward objective risks as soon as possible.

## 6. Conclusion

With the widespread use of Internet platforms, people have more diverse ways to obtain health risk information and have easy and abundant access to information about public health emergencies. The wealth of information affects online users’ perceptions of health risks, and different risk perceptions can lead to differentiated attitudes and behaviors, which in turn bring about risk consequences. This study investigates the information ground factors affecting online users’ perception of health risks in China during COVID-19. The empirical results show that information fluency, information extensibility, platform interactivity, and network connectivity affect online users’ perception of health risks, while information diagnosability, scenario embeddedness, and psychological distance mediate and self-efficacy moderates these relationships. This study is valuable because it provides a new perspective and useful recommendations for online platform managers and risk managers.

## 7. Study limitations

This study also had several limitations, given that different types of public health emergencies exhibit certain differences in their involved populations, hazard characteristics, and that this study was conducted in a Chinese scenario. Future studies will need to identify the mechanisms that affect online users’ perceived health risks in different types of public health emergencies and different cultural contexts and conduct cross-cultural comparative studies.

## Data availability statement

The original contributions presented in the study are included in the article/Supplementary materials, further inquiries can be directed to the corresponding author.

## Author contributions

SH was responsible for data analysis and manuscript writing. QY was responsible for conceptualization. CZ, GC, and HS were responsible for formal analysis, and all of us were jointly responsible for the data coding. All authors contributed to the article and approved the submitted version.

## Funding

This work was supported by the National Natural Science Foundation of China (grant number 71974102).

## Conflict of interest

The authors declare that the research was conducted in the absence of any commercial or financial relationships that could be construed as a potential conflict of interest.

## Publisher’s note

All claims expressed in this article are solely those of the authors and do not necessarily represent those of their affiliated organizations, or those of the publisher, the editors and the reviewers. Any product that may be evaluated in this article, or claim that may be made by its manufacturer, is not guaranteed or endorsed by the publisher.

## Footnotes

^1^ https://www.ibm.com/spss

^2^ www.smartpls.com
