# Supplementary material for: How do online users perceive health risks during public health emergencies? Empirical evidence from China
Source: Front Psychol. 2023 Jan 27;14:1087229. doi: 10.3389/fpsyg.2023.1087229 (PMC9912773; doi:10.3389/fpsyg.2023.1087229)
Supplement: Supplementary file 1 [file Table_1.DOCX]

**Questionnaire**

| **Latent variable** | **code** | **Measuring question** |
| --- | --- | --- |
| Information fluency  （PIF） | PIF1 | I think the information organization and expression of the above materials are very simple |
|  | PIF2 | I think the information organization and expression of the above materials are very fluent |
|  | PIF3 | I think the information organization and expression of the above materials are very clear |
|  | PIF4 | I think the above materials are easy to understand |
| Information extensibility  (PIS) | PIS1 | I think the information in the above materials is very specific |
|  | PIS2 | I feel that the information in the above materials is very rich |
|  | PIS3 | I feel the information in the above materials is very vivid |
|  | PIS4 | I feel that the information in the above materials is rather general and abstract |
| Information diagnosabilityity  (PID) | PID1 | I think the information of the above material types can match my information needs |
|  | PID2 | I think the information of the above material types is reasonable in content |
|  | PID3 | I think the information of the above material types is helpful for me to judge the risk of public health emergencies |
| Platform interactivity  (PIA) | PIA1 | On this platform, I can easily publish information, like, forward, comment and other interactive behaviors |
|  | PIA2 | On this platform, I can easily see the information, likes, forwarding, comments, etc. published by other users |
|  | PIA3 | On this platform, I can easily and timely interact with other users |
| Network connectivity  (NC) | NC1 | I interact with users on this platform for a long time |
|  | NC2 | I interact frequently with users on the platform |
|  | NC3 | I am familiar with users on the platform |
|  | NC4 | I have a close relationship with users on the platform |
| Scenario embeddedness(PE) | PE1 | I think the above materials show the specific and real scene of the public health emergency |
|  | PE2 | The above materials let me experience the scene of the public health emergency |
|  | PE3 | The above materials remind me of the scenes and pictures similar to this public health emergency |
|  | PE4 | The above materials let me imagine the scenes and pictures that may be related to the public health emergency |
| Psychological distance  (PD) | PD1 | The scenes shown in the above materials make me feel that the public health emergency has a strong relationship with me |
|  | PD2 | The scenarios shown in the above materials let me contact my own situation and risk situation |
|  | PD3 | The scenes shown in the above materials make me feel that the public health emergency is very close to me |
|  | PD4 | The scenes shown in the above materials make me feel that the public health emergency is very close to me |
|  | PD5 | The situation shown in the above materials makes me think that the public health emergency is likely to occur in my area |
| Risk perception  (PRB) | PRB1 | I think I'm very likely to be infected with novel coronavirus |
|  | PRB2 | I think the consequences of catching novel coronavirus are very serious |
|  | PRB3 | I'm worried about catching novel coronavirus |
|  | PRB4 | I think the harm of novel coronavirus is controllable |
| Self-efficacy  (SE) | SE1 | If I try my best, I can always solve the problem |
|  | SE2 | Even if others oppose me, I still have a way to get what I want |
|  | SE3 | It's easy for me to stick to my ideals and achieve my goals |
|  | SE4 | I am confident that I can deal with any unexpected event effectively |
|  | SE5 | With my intelligence, I can certainly cope with unexpected situations |
|  | SE6 | If I make the necessary efforts, I will be able to solve most problems |
|  | SE7 | I can face difficulties calmly, because I trust my ability to deal with problems |
|  | SE8 | When faced with a difficult problem, I can usually find several solutions |
|  | SE9 | When I'm in trouble, I can usually think of some ways to cope |
|  | SE10 | I can handle whatever happens to me |
